# Is there more than meets the eye in PSMA imaging in prostate cancer with PET/MRI? Looking closer at uptake time, correlation with PSA and Gleason score

**DOI:** 10.1186/s41824-023-00166-5

**Published:** 2023-04-17

**Authors:** Borjana Bogdanovic, Esteban L. Solari, Alberto Villagran Asiares, Sandra van Marwick, Sylvia Schachoff, Matthias Eiber, Wolfgang A. Weber, Stephan G. Nekolla

**Affiliations:** Department of Nuclear Medicine, School of Medicine, Klinikum Rechts Der Isar, Technical University of Munich, Ismaninger Str. 22, 81675 Munich, Germany

**Keywords:** Whole-body PET, MR, Late dynamic imaging, PET quantification, Prostate cancer, PSMA

## Abstract

**Background:**

In patients with increasing PSA and suspicion for prostate cancer, but previous negative biopsies, PET/MRI is used to test for tumours and target potential following biopsy. We aimed to determine different PSMA PET timing effects on signal kinetics and test its correlation with the patients’ PSA and Gleason scores (GS).

**Methods:**

A total of 100 patients were examined for 900 s using PET/MRI approximately 1–2 h p.i. depending on the tracer used (^68^Ga-PSMA-11, ^18^F-PSMA-1007 or ^18^F-rhPSMA7). The scans were reconstructed in static and dynamic mode (6 equal frames capturing “late” PSMA dynamics). TACs were computed for detected lesions as well as linear regression plots against time for static (SUV) and dynamic (SUV, SUL, and percent injected dose per gram) parameters. All computed trends were tested for correlation with PSA and GS.

**Results:**

Static and dynamic scans allowed unchanged lesion detection despite the difference in statistics. For all tracers, the lesions in the pelvic lymph nodes and bones had a mostly negative activity concentration trend (78% and 68%, resp.), while a mostly positive, stronger trend was found for the lesions in the prostate and prostatic fossa following RPE (84% and 83%, resp.). In case of ^68^Ga-PSMA-11, a strong negative (*R*_min_ = − 0.62, *R*_max_ = − 0.73) correlation was found between the dynamic parameters and the PSA. ^18^F-PSMA-1007 dynamic data showed no correlation with PSA, while for ^18^F-rhPSMA7 dynamic data, it was consistently low positive (*R*_min_ = 0.29, *R*_max_ = 0.33). All tracers showed only moderate correlation against GS (*R*_min_ = 0.41, *R*_max_ = 0.48). The static parameters showed weak correlation with PSA (*R*_min_ = 0.24, *R*_max_ = 0.36) and no correlation with GS.

**Conclusion:**

“Late” dynamic PSMA data provided additional insight into the PSMA kinetics. While a stable moderate correlation was found between the PSMA kinetics in pelvic lesions and GS, a significantly variable correlation with the PSA values was shown depending on the radiotracer used, the highest being consistently for ^68^Ga-PSMA-11. We reason that with such late dynamics, the PSMA kinetics are relatively stable and imaging could even take place at earlier time points as is now in the clinical routine.

## Introduction

Prostate cancer (PCa), persisting at the top of the global rankings of cancer incidence in men (Sung et al. 2021), has been an almost inexhaustible topic of research in the last decades. Both for PCa diagnostics and treatment monitoring, positron emission tomography (PET) has proven to be an immensely valuable clinical imaging modality, particularly with the introduction of, first, ^68^Ga-, and soon after, ^18^F-labelled tracers incorporating the prostate-specific membrane antigen (PSMA) (Mease et al. 2013). Its ability to detect local tumour and metastases proved superior compared to tracers like choline and fluorodeoxyglucose (FDG) and has led to rapid world-wide adoption and further clinical evaluation (Afshar-Oromieh et al. 2014).

The launch of truly simultaneous hybrid scanners combining PET and magnetic resonance imaging (PET/MRI) opened a new door for PCa PSMA imaging. Combining molecular/functional PSMA expression data together with anatomical data acquired with a very high soft-tissue contrast (significantly higher compared to computed tomography) found its place in those clinical applications where small, potentially pathological soft-tissue structures are investigated. Whether for intraprostatic pathologies, extraprostatic extensions, seminal vesicle involvement, or early changes in the bone PSMA PET/MRI has shown potential for improving diagnostic and prognostic performance in primary PCa as well as PCa biochemical recurrence (Eiber et al. 2016; Guberina et al. 2020).

One important clinical PET/MRI application using PSMA ligands is its use after previous negative biopsies in patients with high suspicion of PCa, implied by a sudden or drastic increase in prostate-specific antigen (PSA) in their blood. In those patients with increasing PSA, PSMA PET/MRI is used to (a) assess the likelihood of tumour presence, and (b) to target potential following biopsy. Thus, reliable and accurate delineation and quantification of detected “hot” spots are crucial for the assessment of potential lesions. To this end, we should be aware at all times of influencing factors like tracer kinetics through the lesions during the acquisition, the post-injection timing of the acquisition itself, or even motion corruption. However, in the clinical routine, dynamic scans tend not to be popular as they usually mean extra scanner time per patient, and thus a lower patient throughput. The acquired PET data are therefore most commonly reconstructed into a static PET image, and the abovementioned factors are often either neglected, assumed negligible, or, in a certain context, taken for granted.

For instance, ^68^Ga- or ^18^F-labelled PSMA agents are usually injected as an intravenous bolus after approximately 60 min of uptake time before the start of the PET imaging with a time corridor of 45 to 100 min (Fendler et al. 2017). Potential delayed imaging bases on initial observations in parallel to effects known from FDG PET without any further thorough evidence of its clinical use. This delay post-injection is indeed solely a recommendation, which was, to the best of our knowledge, in the absence of solid evidence, borrowed from the previous procedure guidelines for FDG PET imaging protocols and, as such, accepted solely because of logistical considerations for the timing of the PET protocol with respect to the delay after the injection time.

While the early dynamic uptake of PSMA has already been a focus of a few study groups (Uprimny et al. 2017a; Schmuck et al. 2017; Olde Heuvel 2021; Sachpekidis et al. 2018; Barakat et al. 2020), the late dynamics have not yet been assessed, to the best of the authors’ knowledge. The PSMA uptake at later time points has been evaluated only in the form of static scans (Schmuck et al. 2017), therefore not investigating the signal kinetics at that later stage, its potential added value for understanding the PSMA accumulation and clearance in different lesions, or even potential correlations of those behaviours with other available clinical parameters.

Fortunately, using a simultaneous PET/MR system, a 15-min-long, high-count (i.e. statistically rich) PET acquisition is possible during the conduction of dedicated MR sequences both for biopsy targeting as well as detection of local recurrence without any additional time for the patient. This 15-min PET acquisition can also be reconstructed into a late dynamic study of the prostate scanning range, potentially providing physicians with additional information without increasing the examination duration. This approach allows the analysis of potential effects coming from different timings of PSMA PET acquisition on PET signal kinetics.

Hence, the aim of our retrospective analysis was to investigate the temporal stability of the PSMA PET signal in the prostate throughout this late dynamic acquisition to provide further rationale for current PSMA PET protocols or to propose different approaches. Moreover, we sought to analyse whether incremental information is hidden in the late dynamic prostate PET data and whether correlations between PSMA PET time activity curves (TACs) and PSA values and Gleason scores (GS) are present.

Furthermore, as the majority of previously conducted dynamic studies (with or without comparison with later static scans) focused on ^68^Ga-labbeled PSMA agents, and only one study addressed the early dynamics of ^18^F-labelled PSMA tracer (Sachpekidis et al. 2019), there was no data on late uptake of PSMA tracers labelled with ^18^F and no chance of comparison. Therefore, acknowledging that the different clearance routes of ^68^Ga (renal) and ^18^F (hepatic) could further influence their late kinetics, this study also focused on seeking out these possible deviations by comparing ^68^Ga-labelled vs. ^18^F-labelled PSMA available clinical tracers.

## Materials and methods

### Imaging acquisition

With three important clinical radiotracers for PCa on our disposal, ^68^Ga-PSMA-11, ^18^F-PSMA-1007, and ^18^F-rhPSMA7, we assessed all three in our retrospective analysis. We included one hundred male patients who had PCa lesions in the pelvis and were examined consecutively most recently prior to the beginning of the study. The patients were aged 66 ± 16 years, with median PSA of 7.4 and median GS of 7. They were all examined in one bed position covering the pelvis for 900 s using a clinical 3T PET/MR hybrid system (Biograph mMR, Siemens Healthcare, Erlangen, Germany) (Delso et al. 2011). Forty patients were injected with 105 ± 11 MBq of ^68^Ga-PSMA-11 and were scanned 58.8 ± 12.1 min post-injection (p.i.); eighteen received 321 ± 46 MBq of ^18^F-PSMA-1007 and were scanned 104.1 ± 22.4 min p.i.; the final forty-two patients were given 323 ± 57 MBq of ^18^F-rhPSMA and were scanned 72.2 ± 8.4 min p.i. In total, 28 out of 100 PSMA PET/MRI acquisitions were performed for the purposes of prostate biopsy planning, 26 acquisitions were performed as a part of the PCa primary staging protocol, while the remaining 46 were a part of the PCa biochemical recurrence protocol. The institutional review board of the Technical University Munich approved the retrospective analysis (permit 5665/13 for ^68^Ga-PSMA-11, permit 257/18S for ^18^F-PSMA-1007, and 290/18S for ^18^F-rhPSMA7).

### PET image reconstruction

The 900-s acquisitions were split into 6 frames of 150 s and reconstructed with the standard console reconstruction tool (RetroRecon card) in the PET/MR hybrid system using ordinary Poisson ordered-subsets-expectation maximization (OP-OSEM) iterative reconstruction algorithm with 3 iterations and 21 subsets, matrix size 172 × 172, zoom 1, filtered with a 2-mm FWHM Gaussian smoothing kernel. Additionally, all images were reconstructed in static mode as well, using the same parameters.

### PET image analysis

Qualitative evaluation of the reconstructed PET images was performed by experienced nuclear medicine specialists, who identified all PCa lesions in the pelvic region. For the purposes of quantitative evaluation, for each lesion located in the pelvis, percent injected dose per gram of tissue (%ID/g), mean standard uptake values (SUV_mean_), and mean standard uptake values normalized to lean body mass (SUL_mean_) were calculated for all frames, as well as the respective SUV_mean_ in static mode. TACs were computed, along with the slopes of the linear regression plots against time for the acquired concentration activity in Bq/ml, SUV_mean_, SUL_mean_, and %ID/g data. Finally, Pearson correlation (R coefficient) was tested between all the computed slopes and the corresponding patient’s PSA values and Gleason scores (GS).

## Results

### Qualitative assessment

**Fig. 1:**
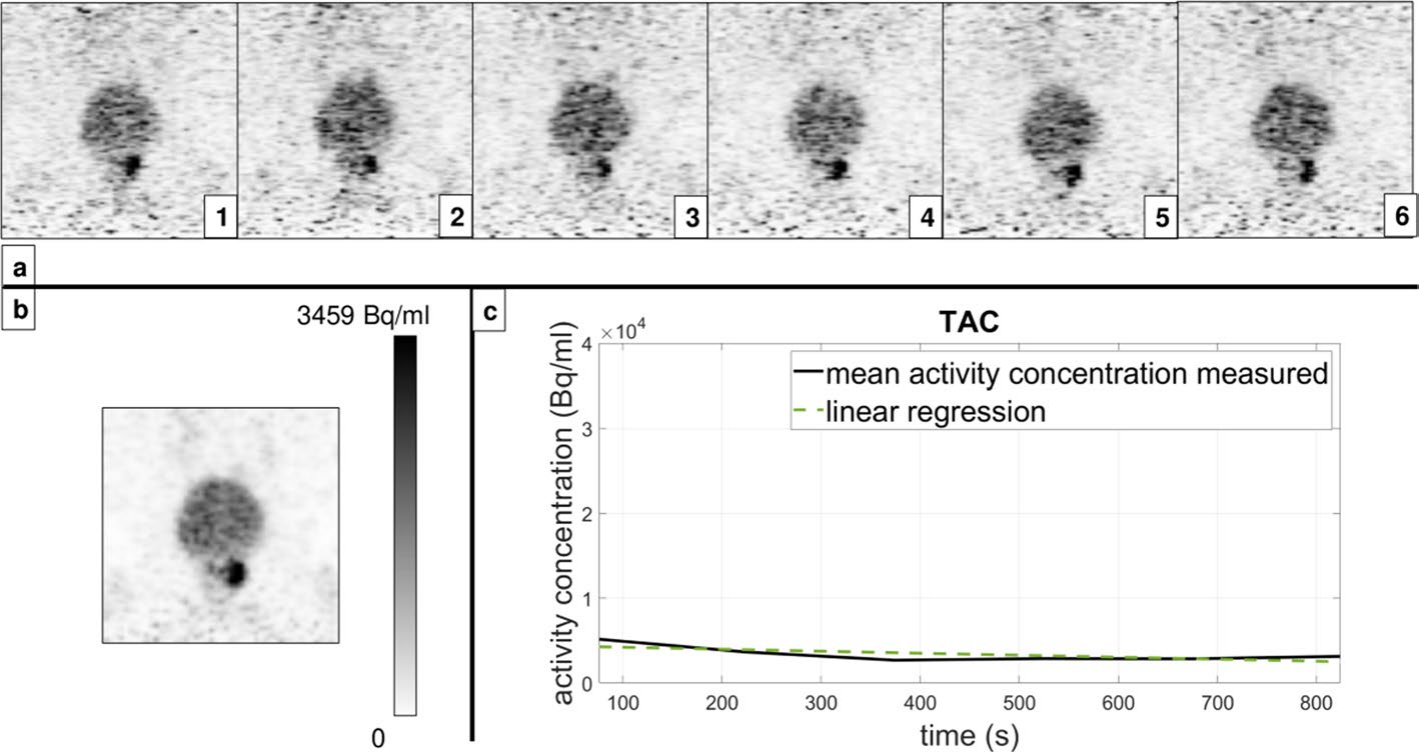
^68^Ga-PSMA-11 PET images (patient 22) of the prostate scanning range reconstructed in dynamic mode (**a**) and static mode (**b**) with the corresponding TAC (**c**)

**Fig. 2:**
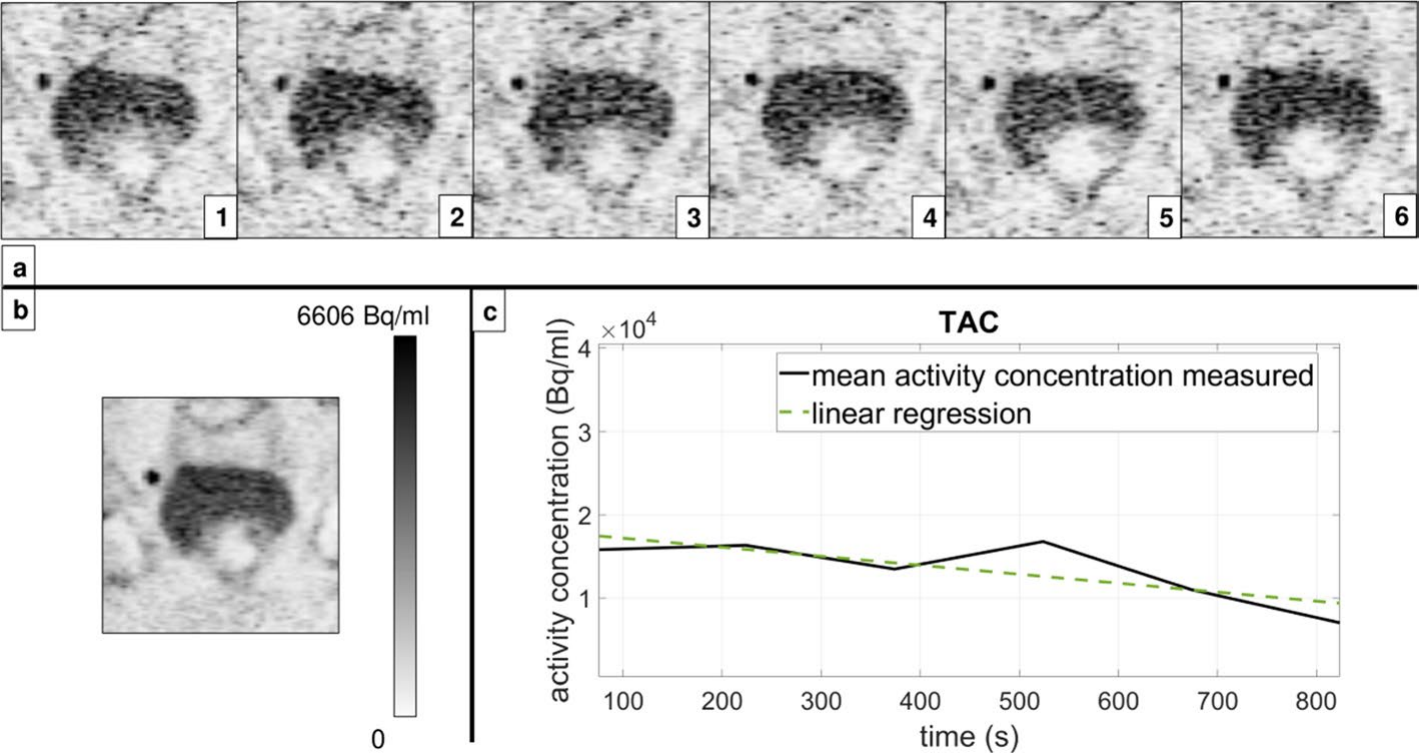
^18^F-PSMA-1007 PET images (patient 51) of the prostate scanning range reconstructed in dynamic mode (**a**) and static mode (**b**) with the corresponding TAC (**c**)

A total of 120 lesions were analysed (80 patients with only one lesion and 20 patients with multiple lesions where two would be randomly chosen and analysed). Among the analysed lesions, 43 were found in the prostate itself (median lesion volume 597 mm^3^), 28 in the pelvic lymph nodes (internal and external iliac, presacral, paravesical, and right obturator; median lesion volume 344 mm^3^), 23 in the prostatic fossa following radical prostatectomy (i.e. RPE; median lesion volume 418 mm^3^), 1 in the penis root (3607 mm^3^), and 25 osseous metastases, where 15 in the pubic bone (median lesion volume 381 mm^3^), 7 in acetabulum (median lesion volume 375 mm^3^), and 3 in ilium (lesion volume 245 mm^3^). Upon qualitative assessment of all three radiotracer datasets (^68^Ga-PSMA-11, ^18^F-PSMA-1007, and ^18^F-rhPSMA7), static and dynamic reconstructions did not differ in the detection of the lesions, i.e. no new lesions were detected in either mode and none were missed either. We report that the diagnostic accuracy was unaffected despite the lower number of counts per frame compared to the static mode, which rendered late dynamic images considerably noisier. We should also note that, upon visual evaluation, motion was detected among 37 of the 120 analysed lesions during the 900 s of acquisition. An example of the reconstructed PET images of the prostate scanning range assessed with ^68^Ga-PSMA-11 is given in Fig. 1 (dynamic and static reconstruction shown in sections (a) and (b), respectively; dynamic reconstruction depicted with 6 time frames; the corresponding TAC depicted in (c) section). Equivalently, an example of the reconstructed PET images of the prostate scanning range assessed with one of the ^18^F-labelled tracers, ^18^F-PSMA-1007, is given in Fig. 2 (an additional figure was not committed to the second ^18^F-labelled tracer to avoid redundancy).

### Quantitative assessment

Quantitative evaluation of the PET images reconstructed in dynamic mode confirmed the results of the qualitative analysis—the activity concentration in all lesions, and thus the resulting SUVs were noticeably affected by noise and had to be smoothed for noise reduction pre-analysis. The influence of the different clearance paths and rates for the three radiotracers was visible in the TACs, especially when it comes to the activity accumulation in the urinary bladder and its frequently resulting “halo” or photopenic zone (i.e. the extinction of PET signal around and due to the high activity concentration in a specific region due to overcorrecting scatter). Namely, the ^68^Ga-PSMA-11 scans featured much more scatter around the bladder and were thus mostly reconstructed using scatter correction with absolute instead of relative scaling, as would have been the standard scenario (in either case, the corresponding static images were reconstructed using the same scatter correction method as the dynamic ones).

**Fig. 3:**
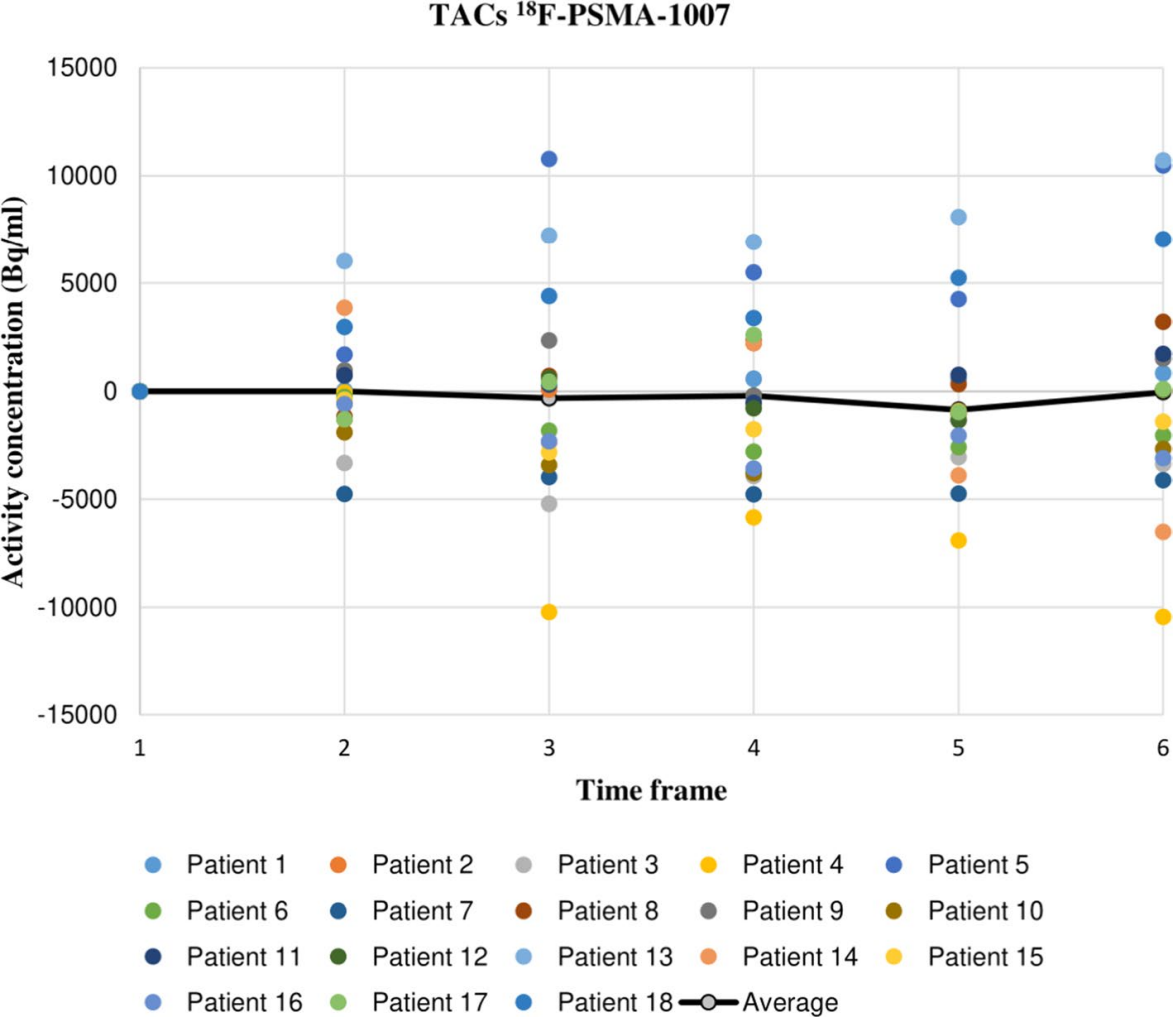
^18^F-PSMA-1007 time-activity curves, normalized from the baseline “zero” time

Upon computing the TACs for all three radiotracers, the lesions found in the pelvic lymph nodes and bones revealed a mostly negative activity concentration trend over time (22 out of 28 and 17 out of 25, respectively), while a mostly positive, often stronger trend over time was revealed for the lesions found in the prostate and prostatic fossa following RPE (36 out of 43 and 20 out of 24, respectively). The lesion found in the penis root also showed stronger, positive trend over time, but was excluded from other groups as it was singular in this patient cohort. An overview of all other analysed lesions sorted by detection site and their dynamics trends is given in Table 1. In majority of the cases, however, the resulting differences in activity over the 900 s of acquisition were comparatively low, thus rendering the signal amplitude relatively stable. For a better overview of the variety in the computed TAC slopes within a single radiotracer subgroup, the TACs for ^18^F-PSMA-1007 dataset are depicted normalized from the baseline “zero” time in Fig. 3, as this radiotracer subgroup counts noticeably fewer patients than the other two and the results can thus be more clearly and reasonably illustrated. Nevertheless, both the ^18^F-rhPSMA7 and ^68^Ga-PSMA-11 dataset feature a comparable variety in TAC slopes. Maximum and mean SUV_max_ difference after the of 15 min were, respectively, 25% and 11% in the ^18^F-rhPSMA7 dataset, 63% (lymph node lesions showed the highest differences overall) and 7% in the ^68^Ga-PSMA-11 dataset, and 35% and 15% in the ^18^F-PSMA-1007 dataset.

**Table 1:** Overview of all analysed lesions sorted by detection site and their dynamics trends

Detection site	Positive trend	Negative trend	Dominant trend (%)
Prostate	36	7	Positive (84%)
Lymph nodes	6	22	Negative (78%)
Post-RPE prostatic fossa	20	4	Positive (83%)
Bones	8	17	Negative (68%)

### Correlation assessment

PSA values were collected from all 100 patients, while temporally matching GS values were available only for 20 patients, as biopsies had either not been performed or documented by that time point, or the patients had already undergone RPE (or PCa therapies) prior to the acquisitions analysed in this study with no indication of new histological analyses since.

The results of the Pearson correlation tests are shown in Table 2. Within the ^68^Ga-PSMA-11 dataset, the correlation between all four dynamic parameter slopes and the PSA values was mostly strong and negative (*R*_min_ = − 0.62, *R*_max_ = − 0.73), though only moderate and again negative against the GSs (*R*_min_ = − 0.42, *R*_max_ = − 0.46). Within the ^18^F-PSMA-1007 dataset, the computed slopes showed almost no correlation with the respective PSA values (*R*_min_ = 0.05, *R*_max_ = 0.08) and moderate positive correlation against the respective GSs (*R*_min_ = 0.43, *R*_max_ = 0.48). Within the ^18^F-rhPSMA7 dataset, the correlation between all four dynamic parameter slopes and the respective PSA values was consistently low positive (*R*_min_ = 0.29, *R*_max_ = 0.33) Similar to the previous two radiotracers, only a moderate positive correlation against the respective GSs was found (*R*_min_ = 0.41, *R*_max_ = 0.44). Looking at the SUVs computed from the static images, we found weak correlation with PSA for all three radiotracers (*R*_min_ = 0.24, *R*_max_ = 0.36) and almost no correlation with the GSs (*R*_min_ = − 0.13, *R*_max_ = 0.19).

**Table 2:** Correlation coefficients (*R*) of the computed PSMA TACs with prostate-specific antigen (PSA) values and Gleason scores

R	Bq/ml		SUV_mean_		SUL_mean_		%ID/g		Static SUV_mean_	
	PSA	GS	PSA	GS	PSA	GS	PSA	GS	PSA	GS
^68^Ga-PSMA-11	− 0.73	− 0.46	− 0.62	− 0.42	− 0.66	− 0.45	− 0.71	− 0.46	0.36	0.19
^18^F-PSMA-1007	0.05	0.48	0.08	0.43	0.08	0.44	0.07	0.46	0.24	− 0.13
^18^F-rhPSMA7	0.29	0.44	0.31	0.41	0.30	0.42	0.33	0.43	0.35	0.17

## Discussion

In this retrospective analysis, we investigated the potential value of reconstructing the 900 s of PSMA PET acquisition of the prostate scanning range into a late-dynamic series. In this context, we evaluated three important clinical PSMA radiotracers, as well as the possible correlation of the generated PSMA time activity curves (TACs) with the patients' PSA values and Gleason scores (GS). Additionally, we questioned the kinetics of the PSMA PET signal in the prostate throughout this late dynamic acquisition in order to evaluate the adequacy of the PSMA PET protocol for the purposes of biopsy planning, primary staging and chemical recurrence detection in prostate cancer.

As previously mentioned, while the potential of early dynamic PSMA PET has already been thoroughly explored in the context of ^68^Ga-PSMA-11 (Uprimny et al. 2017a; Schmuck et al. 2017; Olde Heuvel 2021; Sachpekidis et al. 2018; Barakat et al. 2020) and even investigated for ^18^F-PSMA-1007 in one study (Sachpekidis et al. 2019), to the best of the authors’ knowledge, late PSMA kinetics has not been a topic of any research so far, regardless of the type of the PSMA tracer. Given the lack of literature in this domain, we were curious about all the possible information we could extract as the added value of this PET acquisition already present and approved in the clinical routine. Hence, as PSA values and GS have already been linked to the intensity of the PSMA uptake in PCa patients (Uprimny, et al. 2017; Pereira, et al. 2018), we also looked for a correlation potentially hidden in the dynamic PSMA uptake data.

Despite the increased noise (as seen in Figs. 1 and 2) caused by fewer counts contributing to the image statistics present in the dynamic images, all lesions detected in static mode with high count statistics were discernible in dynamic mode as well. As both static and dynamic modes included the counts from the same time period post-injection, we were not interested in searching for new lesions (like it would often be the case with early dynamic or additionally delayed imaging (Uprimny et al. 2017a; Uprimny et al. 2017b; Hoffmann et al. 2020)), but rather for a “slow-motion” close-up of the lesions already detected in the static images.

The second factor discernible from the dynamic images was fluctuations in activity concentration over selected regions of interest (ROIs) throughout the six time frames (as seen in Fig. 3). In 37 out of 120 cases, the reason for these more prominent fluctuations was also motion, presumably gross patient motion rather than muscle relaxation, as motion was present usually only in one or two non-adjacent time frames and the lesion displacement did not have a gradual, consistent course. The reasons for gross patient motion could be many, however, the usual suspect are more often than not lengthy PET scans which increase patient discomfort over the course of acquisition and may lead to sudden repositioning of the patient. Indeed, in 29 out of 37 cases of detected motion, the patients had a previous partial body scan covering four of five bed positions (neck to pelvis or head to pelvis, respectively) and had to lay still for minimum 30 min prior to the 15-min PET scan.

In addition to this, we noticed the differences in the PSMA TACs fluctuations and their overall trends revealed a pattern depending on the location of the analysed lesion. Interestingly, the patterns were consistent between the three different radiotracers included in this study, despite their different clearance paths and rates. Furthermore, they were consistent between both of the different protocols assessed, i.e. both in patients who were yet to be potentially diagnosed with PCa and have their first staging done, and those assessing chemical recurrence of previously diagnosed PCa and undergoing potential restaging.

While the negative overall trends in the PSMA TACs computed from the activity concentration measured in the lymph nodes could mean that the lymphatic drainage is affecting the activity retention times, this phenomenon has not yet been accounted for in the literature and is yet to be understood. On the other hand, the prostatic lesions and the lesions in the prostatic fossa following RPE mostly showed a tendency to retain activity longer and to still be gaining in activity by 90–125 min p.i. It should be noted that the positive trends in the majority of cases also coincided with larger lesion sizes, which could be understood as a reflection of a higher concentration of PSMA receptors in these larger pathological regions gradually binding more PSMA as it circulates throughout the body over time. The remaining lesion locations were not present with a sufficient number of instances in this patient cohort, which prevented us from drawing more accurate conclusions from their statistics.

One further limitation of this study in terms of accuracy is the unavoidable ambiguity behind the GS values, which could have affected our correlation analysis as well. As previously described in the literature (Wright et al. 2009; Mahal et al. 2016), GS values could be misleading when grading different biopsies, the histology of which can be different, rendering their prognosis different as well, and yet the same GS would be used as a clinical molecular risk factor. For this reason, the accuracy of our GS correlation analysis with the generated TACs is limited by the very method of risk stratification.

With the PSA values, however, while we do not have histological ambiguity, other factors may influence the PSA amount measured such as the age of the patient, the size of the prostate, different kinds of secondary conditions such as inflammations or infections, etc. These factors, nevertheless, do not necessarily affect the PSMA binding properties and activity retention and the variation they introduce in the final PSA value may thus affect the accuracy of our PSA correlation analysis with the generated TACs as well.

Regarding the significant differences in the strength of correlation between the PSA values and the TACs slopes when the three evaluated radiotracers are compared, a few points are brought up for discussion. Namely, the strong correlation of the “late” ^68^Ga-PSMA-11 kinetics with the PSA values could be a result of its particular renal clearance route and the resulting activity retention in the kidneys affecting the activity clearance rate. As opposed to this ^68^Ga-labelled radiotracer, the two ^18^F-labelled ones both have a hepatic clearance route and thus a different pattern of activity retention in the liver, which could be a factor contributing to the results found in this study showing their weak to non-existent correlation with the PSA values.

Another limiting factor which could cause this difference in correlation results as well is the uptake time allowed to the radiotracers before the beginning of the acquisition. Within our patient cohort, ^68^Ga-PSMA-11 had the shortest post-injection acquisition time on average (58.8 ± 12.1 min p.i.), which together with its clearance pattern could have contributed to the stronger correlation results. On the other hand, ^18^F-PSMA-1007 and ^18^F-rhPSMA7 were injected 104.1 ± 22.4 min and 72.2 ± 8.4 min p.i., respectively, which together with their clearance patterns could be the deciding factor for their low correlation results.

On a similar note, the effects of the PSMA PET protocol timing on the signal kinetics during those 15 min of acquisition were a topic of interest as well. As seen from the results, in most of the cases, however, the measured activity concentration signal, whether it be ^68^Ga- or ^18^F-labelled PSMA, featured no drastic amplitude changes. The recommendation on the time given for the PSMA uptake was indeed not strictly followed with each patient in this patient cohort due to the clinical logistical matters, but in the light of these results, the procedure guidelines for the timing of these PET imaging protocols with respect to the delay after the injection time do seem to render relatively stable results. However, our results also suggest caution and special consideration regarding the different activity accumulation and clearance rates between lesions of different sizes and locations.

## Conclusion

Our results indicate that “late dynamic” PSMA data provide additional insight into the different PSMA kinetics through different lesions. Moreover, we found only a moderate correlation between the late dynamic PSMA PET images of pelvic lesions and the patient’s Gleason scores, but simultaneously, a significantly variable correlation with the patient’s PSA values, depending on the radiotracer used in the examination. Interestingly, the highest correlation with the PSA values was recorded using ^68^Ga-PSMA-11 as radiotracer. While these findings were indeed biased by additional noise and motion, we reason that with such a late dynamic, in fact, the PSMA kinetics are relatively stable and imaging could be—especially using shorter lived isotopes—performed at an earlier time point as is now in clinical routine use.

## Data Availability

The datasets generated and/or analysed during the current study are not publicly available as they contain information that could compromise patients’ privacy, but are available from the corresponding author on reasonable request and in anonymous form.

## Abbreviations

PCa: Prostate cancer
PET: Positron emission tomography
PSMA: Prostate-specific membrane antigen
FDG: Fluorodeoxyglucose
MRI: Magnetic resonance imaging
TAC: Time activity curve
PSA: Prostate-specific antigen
GS: Gleason score
OP-OSEM: Ordinary Poisson ordered-subset-expectation maximization
FWHM: Full width at half maximum
SUV: Standardized uptake value
SUL: Standardized uptake value normalized to lean body mass
%ID/g: Percent injected dose per gram tissue
SNR: Signal-to-noise ratio
ROI: Region of interest
RPE: Radical prostatectomy
